# Current Novel Targeted Therapeutic Strategies in Multiple Myeloma

**DOI:** 10.3390/ijms25116192

**Published:** 2024-06-04

**Authors:** Cindy Hsin-Ti Lin, Muhammad Junaid Tariq, Fauzia Ullah, Aishwarya Sannareddy, Farhan Khalid, Hasan Abbas, Abbas Bader, Christy Samaras, Jason Valent, Jack Khouri, Faiz Anwer, Shahzad Raza, Danai Dima

**Affiliations:** 1 Department of Internal Medicine, Case Western Reserve University, MetroHealth Campus, Cleveland, OH 44109, USA; 2 Department of Hematology-Oncology, Roswell Park Comprehensive Cancer Center, Buffalo, NY 14203, USA; muhammad.tariq@rosewellpark.org; 3 Department of Hematology-Oncology, Cleveland Clinic, Taussig Cancer Institute, Cleveland, OH 44195, USA; ullahf@ccf.org (F.U.); samarac@ccf.org (C.S.); valentj3@ccf.org (J.V.); khourij@ccf.org (J.K.); anwerf@ccf.org (F.A.); razas@ccf.org (S.R.); dimad@ccf.org (D.D.); 4 Department of Hematology-Oncology, UT Southwestern, Dallas, TX 75235, USA; aishwarya.sannareddy@utsouthwestern.edu; 5 Department of Internal Medicine, Monmouth Medical Center, Long Branch, NJ 07740, USA; farhankhalid17@gmail.com; 6 Medical College of Wisconsin, Milwaukee, WI 53226, USA; habbas@mcw.edu; 7 School of Medicine, University of Missouri–Kansas City, Kansas City, MO 64110, USA; abbasbader@mail.umkc.edu; 8 Fred Hutchinson Cancer Center, University of Washington, Seattle, WA 98109, USA

**Keywords:** multiple myeloma, targeted therapy, immunotherapy

## Abstract

Multiple myeloma (MM) is a hematologic malignancy caused by the clonal expansion of immunoglobulin-producing plasma cells in the bone marrow and/or extramedullary sites. Common manifestations of MM include anemia, renal dysfunction, infection, bone pain, hypercalcemia, and fatigue. Despite numerous recent advancements in the MM treatment paradigm, current therapies demonstrate limited long-term effectiveness and eventual disease relapse remains exceedingly common. Myeloma cells often develop drug resistance through clonal evolution and alterations of cellular signaling pathways. Therefore, continued research of new targets in MM is crucial to circumvent cumulative drug resistance, overcome treatment-limiting toxicities, and improve outcomes in this incurable disease. This article provides a comprehensive overview of the landscape of novel treatments and emerging therapies for MM grouped by molecular target. Molecular targets outlined include BCMA, GPRC5D, FcRH5, CD38, SLAMF7, BCL-2, kinesin spindle protein, protein disulfide isomerase 1, peptidylprolyl isomerase A, Sec61 translocon, and cyclin-dependent kinase 6. Immunomodulatory drugs, NK cell therapy, and proteolysis-targeting chimera are described as well.

## 1. Introduction

Multiple myeloma (MM) is the second most common hematologic malignancy in adults, characterized by the abnormal proliferation of clonal plasma cells in the bone marrow. Malignant plasma cells disrupt the bone marrow microenvironment, secrete excessive amounts of nonfunctional monoclonal immunoglobulins, and increase osteoclastic activity, leading to organ damage, with the most frequent manifestations including anemia, renal failure, hypercalcemia, and lytic bone lesions [1,2].

Over the past decades, robust preclinical and clinical research has led to the development of novel therapies that have significantly evolved the treatment landscape of MM [3]. The introduction of these agents into clinical practice has led to prolonged progression-free survival (PFS) and improved overall survival of patients with a reduced treatment toxicity profile [4]. Despite these advances, MM remains an incurable disease with aggressive biology in patients who become resistant to multiple drug classes, highlighting the need for new therapeutic targets and the development of new therapeutic modalities. In this review, we will provide a brief overview of recently approved treatments in MM, discuss novel investigational therapies, and describe promising future approaches and targets.

## 2. B Cell Maturation Antigen (BCMA)

BCMA is a transmembrane glycoprotein of the tumor necrosis factor receptor superfamily expressed on late memory B and plasma cells [5]. In conjunction with a ligand B cell-activating factor (BAFF) and a proliferation-inducing ligand (APRIL), BCMA plays a key role in supporting plasma cell longevity [5,6]. BCMA is utilized as a drug target in myeloma treatment in multiple forms, including chimeric antigen receptor (CAR) T cell therapies, bispecific antibodies, and antibody-drug conjugates (ADCs) [7].

### 2.1. Anti-BCMA Chimeric Antigen Receptor (CAR) T Cell Therapies

CAR T cells are engineered cells that have been genetically altered to express a synthetic receptor that binds to tumor targets [8]. Physiologic T cells are collected from the patient and then manufactured to express transmembrane CARs through the transfer of a gene encoding the CAR construct via a viral vector [9]. CARs consist of multiple domains, including an extracellular single chain variable fragment (scFv) that binds to the target antigen, an intracellular activation domain derived from CD3ζ, and an intracellular costimulatory molecule (typically 4-1BB) that helps to further enhance T cell responses. Two CAR T cell therapy products targeting BCMA have been approved thus far for clinical use, and others are undergoing preclinical and clinical investigation.

#### 2.1.1. Approved BMCA-Directed CAR T Cell Therapies

Idecabtagene vicleucel (ide-cel) and ciltacabtagene autoleucel (cilta-cel) are anti-BCMA autologous CAR T cell therapies currently approved by the US Food and Drug Administration (FDA) for RRMM based on the phase 2 KarMMa-1 and CARTITUDE-1 trials, respectively [10,11]. Both therapies are approved for use after four or more prior lines of therapy, including an immunomodulatory agent (IMiD), a proteasome inhibitor (PI), and an anti-CD38 monoclonal antibody (mAb).

Other studies to determine the safety and efficacy of ide-cel and cilta-cel in earlier lines of therapy are currently ongoing. The KarMMa-3 trial compared ide-cel versus standard regimens in patients with triple-class-exposed RRMM who have received 2–4 prior lines of therapy. The trial showed that median progression-free survival (PFS) was significantly prolonged in the ide-cel group (13.3 months) compared to the standard regimen group (4.4 months) (hazard ratio (HR) 0.49; 95% confidence interval (CI) 0.38–0.65) at a median follow-up of 18.6 months with a toxicity profile similar to that of previous studies [12,13]. The Myeloma CAR T consortium reported similar efficacy and toxicity of ide-cel in a real-world patient population [14], including those with renal insufficiency [15] and frail patients [16]; however, patients who had prior BCMA-directed therapy exposure [17] and patients with extramedullary disease [18] appeared to have worse outcomes.

The phase 2 CARTITUDE-2 trial explored the use of cilta-cel in MM patients after 1–3 prior lines of therapy (cohort A) and with early relapse after first-line treatment (cohort B). Available data show that patients in both cohorts experienced deep and durable responses, with an overall response rate (ORR) of 95% and 100%, respectively, and ≥complete response (CR) rates of >90% in both cohorts [19]. The phase 3 CARTITUDE-4 trial compared cilta-cel with pomalidomide, bortezomib, and dexamethasone or daratumumab, pomalidomide, and dexamethasone in patients with lenalidomide-refractory MM after 1–3 prior lines of treatment. Cilta-cel significantly prolonged PFS when compared to standard of care at a median follow-up of 16 months [20]. Analysis of the as-treated set in CARTITUDE-4 also demonstrated potent efficacy, with a PFS rate of 85% at 12 months post-infusion [21].

#### 2.1.2. Investigational BCMA-Directed CAR T Cell Therapies

Other anti-BCMA CAR T cell products are currently under investigation. CART-Ddbcma binds to BCMA at a unique site called a D domain. D domains are small, predominantly helical proteins lacking disulfide bonds that serve as the targeting domain in lieu of the conventional scFv [22]. A phase 1 study of CART-Ddbcma is currently ongoing, with interim results showing an ORR of 100% (≥CR 76%) and a median PFS of 18 months [23]. An academic product, ARI0002h (cesnicabtagene autoleucel), is also undergoing evaluation in a phase 1/2 study. Ongoing results demonstrate good efficacy with an ORR of 95% [24]. HBI0101 is another academic anti-BCMA CAR T cell therapy shown to have good tolerability and efficacy in a phase 1 dose-escalation study (NCT04720313), with ORR of 85% and CR rate of 71% in the high-dose cohort [25].

Due to the high cost and lengthy manufacturing process of commercially available CAR T products, several efforts to develop novel CAR T manufacturing platforms are also underway. These technologies aim to decrease the vein-to-vein time and expand access to CAR T. Early results appear promising. A phase 1 study of GC012F, a BCMA and CD19 dual targeting CAR T developed on the FasT CAR T platform, enrolled 29 heavily pretreated RRMM patients. The ORR was 93.1%, with most patients achieving very good partial response (VGPR) or better and all patients achieving minimal residual disease (MRD) negativity by flow cytometry. The median duration of response (DOR) was 37 months (95% CI 11.0-nonreached [NR]), and the median PFS was 38 months (95% CI 11.8-NR). Mostly low-grade cytokine release syndrome (CRS) was reported in 86.2% of patients. No immune effector cell-associated neurotoxicity syndrome (ICANS) was observed. Notably, GC012F was detectable in 79.3% of patients at 6 months and in 55.2% at 12 months after infusion [26].

Another phase 1 clinical trial of durcabtagene autoleucel (PHE885), an anti-BMCA CAR T product manufactured using the T-Charge platform, reported a 98% overall response rate (ORR) across all dose levels (2.5–20 × 10^6^ CAR T cells) and 100% ORR at doses >5 × 10^6^ cells [27]. Furthermore, patients who received durcabtagene autoleucel have been shown to maintain long-term persistence of a highly diverse TCR repertoire in CAR T cells [28].

Zevorcabtagene autoleucel (zevor-cel, CT053) is a fully human autologous anti-BCMA CAR T cell that has shown promising results for RRMM in the phase 1/2 studies LUMMICAR-1 and LUMMICAR-2 [29,30]. Zervo-cel demonstrated 100% ORR in both trials with no grade 3 or higher CRS or neurotoxicity events. Orvacabtagene autoleucel (orva-cel, JCARH125) is another second-generation fully human anti-BCMA CAR T cell product that demonstrated good efficacy and safety in heavily-pretreated RRMM patients in the phase 1/2 EVOLVE study [31].

Allogeneic anti-BCMA CAR T cells compose another developing area of anti-BCMA CAR T products and may be a preferable option to autologous CAR T as allogeneic products are readily available and may have the superior cancer-killing capacity [32]. P-BCMA-ALLO1 is an allogeneic anti-BCMA CAR T manufactured from healthy donor T cells using nonviral transposon-based integration that has been shown in a phase 1 trial to have low rates of CRS and ICANS and no observed graft versus host syndrome [33]. It was granted orphan drug designation by the FDA in March 2024 as a potential therapeutic option for RRMM. ALLO-715 is another allogeneic CAR T therapy studied in the phase 1 UNIVERSAL trial. Interim results show a tolerable safety profile with again low rates of high-grade CRS and ICANS. The ORR was 55.8%, and the median DOR was 8.3 months [34].

### 2.2. Anti-BCMA Multispecific Antibodies

**Figure 1:**
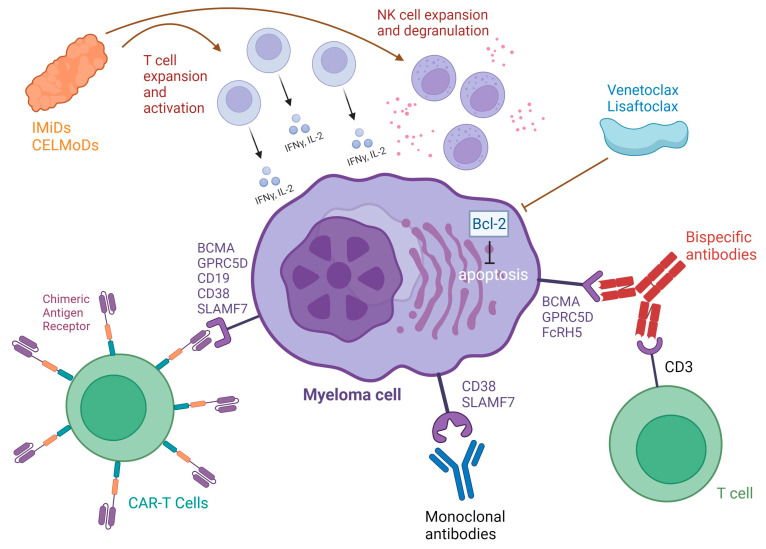
Novel therapies in multiple myeloma and their mechanisms of action. Created with BioRender.com, accessed on 3 June 2024. Abbreviations: IMiD, immunomodulatory drug; CELMoD, cereblon E3 ligase modulator; Th1, Type 1 T helper; NK, natural killer; CAR, chimeric antigen receptor; BCMA, B cell maturation antigen; GPRC5D, G protein–coupled receptor, class C, group 5, member D; IFNγ, interferon γ; TFNα, tumor necrosis factor α; Bcl-2, B cell lymphoma-2; CD18, cluster of differentiation 18; CD38, cluster of differentiation 38, CD3, cluster of differentiation 3; SLAMF7, signaling lymphocyte activation molecular family 7; FcRH5, Fc receptor-homolog 5.

Bispecific and trispecific antibodies are monoclonal antibodies with two or three binding sites that engage with both tumor cells and effector cells (T cells or NK cells) simultaneously, thereby connecting the two and leading to tumor cell destruction through effector cell activation (Figure 1) [35].

#### 2.2.1. Approved Anti-BCMA Bispecific Antibodies

Teclistamab is a bispecific antibody (bsAb) targeting BCMA on MM cells and cluster of differentiation 3 (CD3) on T cells. It was the first bsAb approved for MM based on the results of the phase 1/2 study MajesTEC-1. The study demonstrated an ORR of 63% among 165 patients with five median prior lines of treatment, with 39.4% of patients achieving a CR or better. CRS occurred in 72.1% of patients, but only 0.6% experienced grade 3 CRS, and no occurrences of grade 4 CRS were observed. Three percent of patients developed grade 1 or 2 ICANS [36]. Real-world analyses of teclistamab in patients with heavily pretreated myeloma also show encouraging early efficacy results and good tolerability [37,38].

Elranatamab is another BCMA × CD3 bsAb approved for RRMM based on the phase 2 MagnetisMM-3 trial, where 123 patients with five median lines of therapy were enrolled. The ORR was 61.0%, with 35.0% of patients achieving ≥CR. The median DOR was not reached, and the 15-month DOR rate was 71.5% [39]. There are two ongoing trials investigating the elranatamab in combination with other agents. The MagnetisMM-4 trial (NCT05090566) is a phase 2 study assessing elranatamab in combination with either nirogacestat or lenalidomide/dexamethasone in patients who have received at least three prior therapies, and the MagnetisMM-20 trial (NCT05675449) is a phase 1 study assessing elranatamab in combination with carfilzomib/dexamethasone. Both studies are currently recruiting.

#### 2.2.2. Investigational Anti-BCMA Bispecific Antibodies

Several investigational anti-BMCA bispecific and trispecific antibodies have shown promising results in phase 1 and 2 studies. Linvoseltamab is a BCMA × CD3 bispecific antibody currently under investigation in the LINKER-MM1 trial (NCT03761108). The phase 2 study enrolled RRMM patients who have progressed through three or more lines of therapy, including a PI, IMiD, and anti-CD38 mAb, or were triple-class refractory. The ORR was 71%, with 30% of patients achieving ≥CR. Toxicity occurred in all patients, and 79% of patients experienced a grade ≥3 AE (adverse event); however, high-grade CRS was rare (1% grade ≥3). Infections of any grade occurred in 59.8% of patients, with 36.8% of patients experiencing a grade ≥3 infection [40].

A phase 1 clinical trial is currently investigating the use of F182112, a BCMA × CD3 bispecific antibody, in patients who have received at least two prior therapies, including a PI and IMiD. Preliminary data showed that among the 20 patients evaluated for response, the ORR was 45%, and another 30% had stable disease. Adverse events grade ≥3 occurred in 64% of patients, the most common of which were CRS (72.7%, all grade 12), lymphopenia (68.2%), neutropenia (54.6%), and leukopenia (50%) [41].

Alnuctamab is a 2 + 1 BCMA × CD3 bsAb with bivalent binding to BCMA. A phase 1 trial (NCT03486067) is currently investigating subcutaneous Alnuctamab in patients who have received three or more lines of therapy, including a PI, IMiD, and anti-CD38 mAb. The ORR was 54%, and the median PFS was 10.1 months across all dose levels. All patients who achieved a response and had evaluable MRD samples were MRD-negative (10^−5^ sensitivity by flow cytometry). AEs of any grade occurred in 99% of patients, with grade ≥3 AEs occurring in 81% of patients. The toxicity profile was similar to other bsAb [42].

ABBV-383 is another 2 + 1 BCMA × CD3 bsAb with bivalent binding to BCMA. A phase 1 study investigated ABBV-383 in patients with three or more prior lines of therapy and no prior BCMA exposure. AEs were seen in nearly all patients at all three drug dosages (20 mg, 40 mg, and 60 mg), with 78%, 84%, and 82% of patients experiencing a grade ≥3 event in each respective dosage group. ICANS occurred in 3–5% of patients in each dosage group, with increased severity at higher dosages. There was a high incidence of infection and cytopenias during the treatment period, and treatment discontinuation rates due to AEs were 6%, 11%, and 10%, respectively, in the three groups. ORR was 44%, 64%, and 60%, with 22%, 53%, and 52% of patients achieving ≥VGPR at the 20 mg, 40 mg, and 60 mg dosage groups, respectively (Table 1) [43].

HPN217 is a trispecific antibody that binds to BCMA on MM cells, albumin for half-life extension, and CD3 on T cells. A phase 1 study of HPN217 was recently completed in patients who have received three or more prior therapies, with an ORR of 55%. VGPR or better was noted in 73% of responders, and the median time on treatment was 40 weeks among responders. The therapy was generally well tolerated, with a very low incidence of ICANS and grade ≥3 CRS [44].

A number of bsAb binding to NK cells (instead of T cells) are currently under preclinical investigation. NK-directed treatments are of special interest as evidence suggests that NK cell-based therapies may have a lower level of cytokine release than T cell-based therapies. SAR’514 is a trifunctional molecule that binds BCMA and also activates NK cells through a dual engagement of NKp46 and CD16a. SAR’514 has been shown to promote strong NK cell activity against BCMA+ target MM cells in vitro with very low levels of cytokine release and prolong survival in transgenic mice studies [45].

JNJ-79635322 is a novel BCMA × GPRC5D × CD3 trispecific antibody shown to have robust antitumor activity in in vitro and xenograft models. A phase 1 trial (NCT05652335) on JNJ-79635322 in RRMM is currently ongoing [46].

### 2.3. Anti-BCMA Antibody–Drug Conjugate (ADC)

Belantamab Mafodotin (belamaf) is a novel antibody-drug conjugate against BCMA used in RRMM. Several studies investigating the use of belamaf both as monotherapy and in combination with other agents in NDMM and RRMM are ongoing [47,48,49,50]. DREAMM-8 (NCT03715478) is a phase 3 study comparing the combination of belantamab mafodotin, pomalidomide, and dexamethasone (BPd) versus pomalidomide, bortezomib, and dexamethasone (PVd) as a second line or later treatment for RRMM [51]. A recent press release announced a significant benefit in PFS with BPd as compared to PVd; however, detailed results from the trial are still pending [52]. DREAMM-7 (NCT04246047) is a phase 3 study comparing the combination of belantamab mafodotin, bortezomib, and dexamethasone (BVd) with daratumumab, bortezomib, and dexamethasone (DVd) in RRMM. Results from the trial showed a significant benefit in PFS with BVd as compared to DVd; mPFS in the BVd arm was 36.6 mo (95% CI, 28.4 mo-NR) vs. 13.4 mo (11.1–17.5 mo) in the DVd arm. Ocular AEs were more common in the BVd arm; however, the overall safety profile of BVd was manageable [53].

## 3. G Protein–Coupled Receptor, Class C, Group 5, Member D (GPRC5D)

Beyond BCMA, the G protein–coupled receptor (GPRC5D) is another novel target of interest [54]. GPRC5D is highly expressed on the surface of plasma cells and is also present at low levels of expression in hair follicles and hard keratinizing tissue [55,56]. Its function is not well characterized to date. GPRC5D has been recognized as an immunotherapeutic target in MM that can be effective after antigen escape with BCMA-directed therapies and has been studied in both CAR T cell and bispecific antibody modalities [57].

### 3.1. GPRC5D CAR T Cell Therapy

Several studies on GPRC5D CAR T cell therapies have been completed to date with encouraging results. Mailankody et al. reported a phase 1 dose-escalation study of GPRC5D-targeted CAR T cells (MCARH109). The maximum tolerated dose was identified to be 150 × 10^6^ CAR T cells. At higher doses, 6% of the patients had grade 4 CRS and ICANS, and 11.5% had grade 3 cerebellar disorder. A total of 17 patients were included in the trial, and an ORR of 71% was observed, with a comparable ORR of 70% in the subgroup of patients who had received prior BCMA therapies [57].

A small phase 1 study of GPRC5D-targeted CAR T cells (OriCAR-017) enrolled a total of 10 patients, 50% of whom had previously received anti-BCMA CAR T. An ORR of 100% was noted, with 60% of patients achieving stringent CR and 40% VGPR. Only two patients progressed. No dose-limiting toxic effects or neurologic AEs were observed. Significant toxicities of grade ≥3 included cytopenias. CRS was observed in 90% of patients, but all events were grade 1–2 [58].

A third phase 1 study (NCT04674813) enrolled 70 patients and treated them with another GPRC5D-targeted autologous CAR T product (BMS-986393). Patients who received doses ranging from 75 × 10^6^ to 450 × 10^6^ CAR T cells had an ORR of 86% and CR rate of 38%. In patients treated with prior BCMA-directed therapies, including CAR T, the ORR was slightly lower at 75%. Grade 3-4 AEs were noted in 91% of patients and most commonly involved cytopenias. Four percent of patients developed grade ≥3 CRS, and 11% of patients experienced ICANS-like neurotoxicity (3% grade ≥3). After a median follow-up of 5.6 months, 75% of responses were maintained [59,60].

A phase 2 trial of GPRC5D-targeted CAR T also reported a manageable safety profile and encouraging efficacy. A total of 33 heavily pretreated patients were included in the study, 27% of whom had previous anti-BCMA CAR T. An ORR of 91% was seen, and all patients with prior BCMA therapy achieved at least a partial response. CRS was noted in 76% of patients, but all occurrences were low grade, and neurotoxicity, including grade 2 and 3 ICANS, affected 9% of patients (Table 2) [61].

### 3.2. GPRC5D Bispecific Antibodies

Talquetamab is a bsAb binding to GPRC5D on MM cells and CD3 on physiologic effector cells that was approved for clinical use in RRMM in August 2023 based on the results of the phase 1/2 MonumenTAL-1 study [62]. Recent updated results reported that among patients with prior anti-BCMA CAR T or bsAb, the ORR was 73% and was comparable to the ORR of the overall study population. Furthermore, patients who previously received CAR T (96% anti-BCMA CAR T) had a median DOR of greater than 12.3 months. In contrast, the ORR in those with prior BCMA bsAb was lower at 53%, with lower ORR in patients with a shorter interval between the last dose of bsAb and talquetamab initiation. The median DOR in those with prior BCMA bsAb was also shorter, around six months [63]. Patients who discontinued talquetamab were able to be effectively treated with a variety of subsequent therapies, including CAR T (90% anti-BCMA CAR T) (ORR 67%), anti-BCMA bsAb (ORR 33%), anti-FcRH5 bsAb (ORR 67%), and anti-CD38 mAb-containing regimens (ORR 33%), among others [64].

Talquetamab has also been studied in combination with traditional antimyeloma agents. The phase 1b MonumenTAL-2 study assessed talquetamab in combination with pomalidomide, which yielded an ORR >80%. Weekly dosing of talquetamab appeared superior to biweekly dosing, with 60% of patients achieving ≥CR. Responses were durable, with 100% of responders maintaining their response in both cohorts at 6 months. Furthermore, AEs with the combination were similar to those of the individual agents, and no new or excess additive toxicity was seen. The most common side effects of talquetamab included oral toxicities, dysgeusia in particular, and CRS (mostly grade 1–2). The most common grade 3–4 AEs were cytopenias [65].

The phase 1b RedirecTT-1 trial is a combination study of BCMA- and GPRC5D-targeted bispecific antibodies Teclistamab and Talquetamab and showed a tolerable safety profile consistent with its monotherapy components. The most common AEs were CRS, neutropenia, and anemia. At the recommended phase 2 regimen, the ORR was 92% in patients with advanced RRMM and 83% in patients with extramedullary disease [66].

Forimtamig (RG6234) is a GPRC5D × CD3 bispecific antibody shown to have robust efficacy in a phase 1 study (NCT04557150), with ORR of 71.4% and 60.4% for the intravenous and subcutaneous administration cohorts, respectively. Forimtamig also demonstrated a manageable safety profile with low rates of ICANS and grade ≥3 CRS [67].

## 4. Fc Receptor-Homolog 5 (FcRH5)

FcRH5 is a cell surface antigen with an unknown function that belongs to the immunoglobulin superfamily [68]. It is expressed on mature B cells and plasma cells, with particularly high levels of expression on myeloma cells, and is, therefore, under investigation as a drug target in MM [69].

### 4.1. FcRH5 CAR T Cell Therapy

FcRH5 CAR T cells have been shown to exhibit robust tumoricidal efficacy both in vitro and in murine xenograft models, including in BCMA-deficient cells [70]. Furthermore, FcRH5/BCMA tandem CAR T cells, or CAR T cells with dual anti-FcRH5 scFv and anti-BCMA scFv connected to a single stalk, have been shown to display improved efficacy compared with monospecific CAR T cells in murine xenograft models [70].

### 4.2. FcRH5 Bispecific T Cell Antibodies

Cevostamab is a FcRH5×CD3 bispecific antibody under investigation as a potential therapy for RRMM, particularly in the setting of relapse after BCMA-directed therapies. Since FcRH5 expression occurs independently of BCMA, FcRH5-directed therapies may be beneficial for targeting BCMA low or negative tumor cells and re-invigorating BCMA CAR T cells against residual BCMA-positive tumor cells.

CAMMA-2 is an ongoing phase 1/2 trial evaluating cevostamab monotherapy in patients with triple-class refractory RRMM who have received a prior anti-BCMA agent [71]. Another single-center phase 2 trial is investigating cevostamab as consolidation therapy after anti-BCMA CAR T for RRMM (Table 1) [72]. The results of both studies are eagerly awaited.

## 5. Cluster of Differentiation 38 (CD38)

CD38 is a transmembrane glycoprotein with ectoenzymatic activity that serves multiple functions, including the catabolism of nicotinamide adenine dinucleotide (NAD+) and nicotinamide adenine dinucleotide phosphate (NADP) [73,74]. It is highly expressed in myeloma cells, with lower levels of expression present in other hematopoietic cells and nonhematopoietic tissues. Daratumumab and isatuximab are mAbs against CD-38 and are established agents in the treatment landscape of MM [75]. More recently, there has been an effort to target CD-38 with advanced novel agents.

### 5.1. Anti-CD38 T Cell Engagers

Several anti-CD38 bispecific and trispecific mAbs are undergoing investigation in preclinical and phase 1 studies. Igm-2644 is a novel CD38×CD3 bispecific IgM T cell engager that has demonstrated antimyeloma activity and lower levels of cytokine release and T cell fratricide than bispecific IgG antibodies in in vitro and in vivo studies [76]. A phase 1 trial (NCT05908396) to investigate Igm-2644 in RRMM is currently recruiting.

Trispecific mAbs may help overcome antigen escape by targeting two antigens simultaneously and are also under exploration. SAR442257 is a CD38/CD28×CD3 trispecific mAb that has demonstrated tumoricidal activity in ex vivo and in vitro studies, suggesting that dual CD38/CD28 targeting may be efficacious in RRMM patients previously exposed to anti-CD38 mAb [77]. ISB-2001 is another trispecific mAb that targets both BCMA and CD38 and has shown potent antimyeloma effects in preclinical studies [78]. An ongoing phase 1 trial (NCT05862012) is studying its use in RRMM [79].

### 5.2. Anti-CD38 Antibody–Drug Conjugates (ADCs)

Several anti-CD38 ADCs are currently under investigation. Modakafusp alfa (TAK-573) is an anti-CD38 ADC consisting of two attenuated IFNα2b molecules attached to an anti-CD38 IgG4 mAb that drives preferential IFNα signaling in myeloma cells [80]. Several studies are currently underway to investigate the role of modakafusp in MM, including its use as a single agent in triple-class refractory patients who have undergone three or more lines of therapy (iinnovate-1, NCT03215030) or in combination with daratumumab in RRMM patients (iinnovate-3, NCT05590377). Modakafusp is also being studied in combination with standard-of-care agents as part of double or triplet therapy in RRMM or as maintenance therapy after autologous transplant (ASCT) in NDMM (iinnovate-2, NCT05556616). The phase 1/2 study iinnovate-1 showed that modakafusp might facilitate the activation of both innate and adaptive immune responses. Increased NK cell and peripheral CD8 T cell activation and cytotoxic function, as well as enhanced proliferation of NK cells, dendritic cells, and monocytes in both bone marrow (BM) and peripheral blood (PB) were noted after modakafusp administration. Furthermore, modakafusp use led to increased CD86 expression on PB dendritic cells and monocytes, suggesting enhanced antigen presentation and costimulation ability. Modakafusp also increased CD68 expression in both BM and PB, indicating a proinflammatory M1 macrophage-type response.

MT-0169 (also known as TAK-169) is an investigational engineered toxin body (ETB) comprised of a single-chain variable fragment (scFv) with an affinity for CD38, fused to a ribosome-inactivating Shiga-like toxin-A subunit (SLTA) [81]. Preclinical in vivo studies have shown promising results, including efficacy in patient-derived samples with previous daratumumab exposure, leading to a phase 1 multicenter trial that was recently completed [82]. We eagerly await the results of this trial.

### 5.3. Anti-CD38 CAR and Dimeric Antigen Receptor (DAR) T Cell Therapies

Anti-CD38 CAR T cells have demonstrated robust in vivo activity against CD38+ lines in preclinical studies [83,84,85]. However, clinical evidence of efficacy is currently scarce. A phase 1 trial of CD38-directed CAR T (CAR2 anti-CD38 A2 CAR T cells) in RRMM patients showed at best PR and short durations of response [86]. In contrast to traditional CAR T cells, which utilize a scFv antigen binding region, dimeric antigen receptor (DAR) T cells incorporate a fragment antigen-binding (Fab) region that enhances T cell expansion and efficacy [87]. DART-RRMM-101 is a phase 1b study assessing allogeneic anti-CD38 A2 dimeric antigen receptor (DAR) T cells in RRMM (NCT05007418) with no reported interim results yet (Table 2).

## 6. Cereblon

Cereblon is a component of the E3 ubiquitin protein ligase complex CRL4. When bound to IMiDs, cereblon acquires the ability to target and degrade the transcription factors Ikaros and Aiolos, leading to the inhibition of cell proliferation [88]. In detail, IMiDs are thalidomide analogs derived from glutamic acid that promote cereblon-dependent destruction of Ikaros family proteins, inhibit tumor necrosis factor (TNF) activity, and exert antiangiogenic and anti-inflammatory properties [88,89]. The IMiD agents lenalidomide and pomalidomide are currently used as a standard of care in routine clinical practice. Over the past few years, novel agents similar to IMiDs, known as cereblon-E3 ligase modulating agents (CELMoDs), have developed. CELMoDs chemically resemble IMiDs but have additional phenyl and morpholino moieties that increase their affinity to cereblon and enhance their degradation of Ikaros and Aiolos [90]. The increased potency of CELMoDs allows these agents to exhibit robust antiproliferative properties even in IMiD-resistant samples and cell lines with low cereblon expression [91]. CELMoDs are currently being assessed in the clinical setting.

### Cereblon-E3 Ligase Modulating Agents

Iberdomide is a CELMoD with increased antitumor activity compared to other thalidomide analogs that share a similar mechanism of action [92]. A phase 1/2 study of iberdomide with dexamethasone in RRMM showed an ORR of 26% in the dose expansion cohort with an acceptable safety profile [93]. Another phase 1/2 trial (NCT05199311) explores outcomes of iberdomide with carfilzomib and dexamethasone (KID) in newly diagnosed MM (NDMM) patients who are transplant-eligible. Among the nine out of ten patients with response data available who received 2–4 cycles (median 3 cycles) of KID induction, the ORR was 100%. Six patients proceeded to ASCT, and the ORR was 100% at 3 months post-transplant (50% CR) [94].

Furthermore, a phase 2 clinical trial is exploring the use of iberdomide as post-ASCT maintenance in NDMM. After 6 treatment cycles, response improvement was noted in 48% of patients treated with 1 mg and 45% of patients treated with 1.3 mg of iberdomide [95]. These results suggest that iberdomide maintenance may have superior efficacy at 6 months than lenalidomide maintenance, which showed a deepening of response in 26% of patients at 6 months in a prior study [96]. The EXCALIBER-maintenance study is a randomized controlled phase 3 trial currently enrolled with the aim of comparing iberdomide maintenance to lenalidomide maintenance to further elucidate its role in the maintenance space.

Mezigdomide is another CELMoD that exhibits significant antiproliferative and tumoricidal effects in myeloma cells. A phase 1/2 study of mezigdomide with dexamethasone in heavily pretreated RRMM patients showed an ORR of 41%, a median DOR of 7.6 months, and a median PFS of 4.4 months. AEs mainly consisted of myelotoxicity [97]. Mezigdomide is also studied in combination with either daratumumab–dexamethasone (MDD) or elotuzumab–dexamethasone (MED) in RRMM patients with 2-4 prior therapies and IMiD refractoriness. The MDD cohort had an ORR of 75%, while ORR in the MED cohort ranged from 36% (in the 0.3 mg dose level) to 56% (in the 0.6 mg dose level). Most patients in the latter arm had prior anti-CD38 mAb therapy exposure [98].

## 7. Signaling Lymphocyte Activation Molecular Family 7 (SLAMF7)

SLAMF7 is a receptor highly expressed in MM cells that may act as a growth factor, as suggested by the available literature [99]. SLAMF7 regulates NK cell degranulation and enhances NK cell-mediated tumor clearance [100]. Additionally, the extracellular domain of SLAMF7 can be cleaved to form soluble SLAMF7 (sSLAMF7), a compound that facilitates myeloma cell growth [99]. SLAMF7 expression can be retained in extensively treated RRMM patients, making it a desirable therapeutic target [101]. Elotuzumab is an anti-SLAMF7 mAb that exerts its antitumor effects through multiple mechanisms, including neutralization of sSLAMF7, NK cell-mediated antibody-dependent cellular cytotoxicity (ADCC), macrophage-mediated antibody-dependent cellular phagocytosis (ADCP), and NK cell activation [99]. Elotuzumab is approved for RRMM in combination with lenalidomide–dexamethasone or pomalidomide–dexamethasone [102,103]. Several CAR T therapy products against SLAMF7 have shown promising results in in vitro and in vivo studies [104,105,106].

The ongoing phase 1/2 CARAMBA-1 trial (NCT04499339) aims to assess the outcome of autologous anti-SLAMF7 CAR T cells in RRMM (Table 2) [107].

## 8. B Cell Lymphoma-2 (BCL-2)

The BCL-2 protein family is a group of proteins that consists of prosurvival BCL-2-like proteins, proapoptotic BAX/BAK proteins, and proapoptotic BH3-only proteins [108]. Together, these molecules regulate the intrinsic apoptosis pathway. Disruption of BCL-2-regulated apoptosis drives myeloma cell survival, especially in the t(11;14) genetic subtype, which expresses higher levels of antiapoptotic protein BCL-2 [109]. Though BCL-2 inhibitors have not yet been formally approved by the FDA for use in MM, therapeutics that target this pathway have demonstrated good antimyeloma activity.

Venetoclax is a BH3 mimetic currently used in cancer practice that binds and inhibits prosurvival Bcl-2 proteins (Figure 1). It has shown promising efficacy in phase 1, 2, and 3 trials, both as monotherapy and in combination with dexamethasone, PIs, or CD38 mAbs in patients with t(11; 14) and high BCL2 expression [110,111,112]. Newer BCL-2 agents, such as sonrotoclax (BGB-11417), are currently under investigation and are more potent BH3 mimetics. An ongoing phase 1b/2 trial is investigating sonrotoclax monotherapy, sonrotoclax–dexamethasone, or sonrotoclax–carfilzomib–dexamethasone in RRMM patients who are positive for t(11;14), with promising initial safety and efficacy results [113].

Lisaftoclax (APG-2575) is another selective BCL-2 inhibitor under clinical investigation in both hematologic and solid malignancies. A phase 1b/2 trial (NCT04674514) investigating lisaftoclax both as monotherapy and in combination with lenalidomide and dexamethasone is currently in progress [114]. Another multicenter clinical trial (NCT04942067) investigated lisaftoclax in combination with pomalidomide–dexamethasone or daratumumab–lenalidomide–dexamethasone in patients with RRMM or relapsed–refractory light chain amyloidosis, demonstrating an ORR ranging between 60 and 100% [115].

## 9. NK Cell Therapy

MM cells evade the immune system in part through inhibition of natural killer (NK) cells’ cytotoxic activity against tumor cells [116]. NK cell therapies seek to restore NK cell recognition of myeloma cells and NK cell function.

### 9.1. Autologous Non-CAR NK Cells

The use of autologous NK cells has been investigated both in the RRMM setting (NCT02481934) and as consolidation after ASCT and has thus far shown promising responses [117,118]. A phase 1 study (NCT04558853) of autologous NK cells as a consolidation following ASCT showed that at 6 months post-transplant, 67% of patients achieved CR, 17% achieved VGPR, and 17% experienced relapse. The median PFS was 34 months. No serious AEs or CRS were seen [118].

Antibody recruiting molecules (ARMs) are bifunctional molecules that bind to endogenous antibodies and guide them toward cancer cells and have been utilized to enhance autologous NK cell therapy after ASCT in MM [119]. BHV-1100 is a novel CD38 ARM that enhances NK cell recruitment for antibody-dependent cell cytotoxicity (ADCC) without causing fratricide [120]. A phase 1/2 study investigating BHV-1100 primed autologous cytokine-induced memory-like (CIML) NK cells is currently ongoing. In this study, CIML NK cells were infused 24 h after 200 mg/m^2^ melphalan administration, and interim data show a 3-fold expansion with a peak on day +28 and persistence until day +60. No severe AEs were noted other than anticipated infusion reactions [120].

### 9.2. Nonautologous CAR NK Cells

Multiple nonautologous CAR NK cells are under investigation. Allogeneic BCMA/GPRC5D dual-targeted CAR NK cell therapy has demonstrated efficacy in mice xenografts [121]. Pluripotent stem cell (iPSC)-derived CAR NK cells that cotarget GPRC5D and CD38 (FT555) also appear to be efficacious in combination with daratumumab in mouse models [122]. Additionally, cord blood-derived CD-70 CAR NK cells have been investigated. CD-70 is a protein upregulated on many hematologic and solid cancer cells that has been found to be highly expressed in MM cells of RRMM patients who have progressed on BCMA-directed therapies [123]. Preclinical studies of CD-70 CAR NK cells in mice xenografts demonstrated improved tumor control and increased survival compared to treatment with nontransduced NK cells, including in BCMA knockout mouse models [123]. A phase 1/2 clinical trial (NCT05092451) is currently recruiting to further investigate cord blood-derived CD-70 CAR NK cells for the management of RRMM.

## 10. Kinesin Spindle Protein Inhibitors

Kinesin spindle protein (KSP), also referred to as EG5/KIF11, is predominantly expressed in cells undergoing division and plays a pivotal role in the formation of the mitotic bipolar spindle. KSP inhibition causes the emergence of unusual monopolar spindles and disruption of centrosome separation [124,125,126].

Filanesib, or ARRY-520, is a selective kinesin spindle protein inhibitor (KSPi) that blocks the enzymatic activity of mitotic kinesin KSP and induces prolonged mitotic inhibition. Filanesib, in combination with bortezomib–dexamethasone, has demonstrated good efficacy in RRMM with 1q21 gain and t(11,14). A phase 1 trial investigating the combination of filanesib and bortezomib–dexamethasone showed an ORR of 39% with a median DOR of 14.1 months and a median PFS of 8 months. An ORR of 45% and a median PFS of 9.1 months were seen among patients with 1q21 gain [127,128]. The benefit in patients with 1q21 gain could be attributed to the elevated expression of apoptosis inhibitor myeloid cell leukemia 1 (MCL-1) seen in this subset [129]. Patients with t(11;14) also showed encouraging responses with an ORR of 33.3% and a median PFS of 15 months [130].

## 11. Proteolysis-Targeting Chimera

Proteolysis-targeting chimeras (PROTACs) are molecules that selectively degrade target proteins via the ubiquitin–proteasome system. PROTACs consist of two ligand-binding domains joined by a linker and bind to both E3 ubiquitin ligase and a protein of interest. Simultaneous binding induces ubiquitination and subsequent degradation of the target protein [131,132].

PROTACs have shown promising activity in preclinical studies. In vitro and xenograft models of PROTACs targeting BRD4 and other BET family members have demonstrated time- and concentration-dependent antimyeloma activity associated with decreased MYC and Akt/mTOR signaling, in addition to the ability to overcome resistance to PI and IMiDs [133]. BET-targeted PROTACs were also shown to inhibit cell proliferation in multiple MM cell lines, fresh patient samples, and MM xenografts in another preclinical trial [134]. Another in vitro study tested a newer experimental PROTAC targeting hRpn13, a proteasome substrate receptor upregulated in MM [135].

Not only have studies explored the efficacy and safety of PROTACs, but the resistance mechanisms of PROTACs in myeloma have also been investigated. A recent in vitro and public database study showed that BET-PROTAC resistance in MM cells is mediated by the upregulation of ABCB1 expression and that genetic expression of C1orf112, CCDC167, and CRIP2 may correlate with drug resistance and poor prognosis [136]. Clinical development of PROTACs in the treatment of MM is highly anticipated.

## 12. Protein Disulfide Isomerase 1 (PDIA1)

Protein disulfide isomerases (PDIs) are a group of enzymes located in the endoplasmic reticulum (ER) and involved in the folding and structural integrity of antibodies and other secretory proteins dependent on intramolecular disulfide bond rearrangements for synthesis and stabilization [137,138]. Since malignant plasma cells in MM synthesize large amounts of proteins, myeloma cells are highly sensitive to protein dysregulation, as evidenced by the potent efficacy of PIs.

Several PDI inhibitors have been developed. CCF642 was the first PDI inhibitor developed and exhibited efficacy comparable to that of bortezomib in mouse models [139]. However, CCF642 had poor aqueous solubility, limiting its advancement to clinical development. Similarly, E64FC26 is a pan-PDI inhibitor shown to improve survival and act synergistically with bortezomib in in vivo studies [140]. More recently, the novel PDIA1 inhibitor CCF642-34 was developed. CCF642-34 has been shown to effectively induce myeloma cell death both in vitro and in vivo without affecting healthy hematopoietic stem and progenitor cells. Compared to CCF642, CCF642-34 exhibited improved solubility, selectivity, and potency, making it a promising drug candidate for RRMM [141].

## 13. Peptidylprolyl Isomerase A (PPIA)

PPIA (also known as cyclophilin A) is an enzyme involved in the folding and trafficking of newly synthesized proteins [142]. High levels of PPIA expression have been found in multiple cancers [143]. One single-arm phase 2 trial enrolled 41 NDMM patients who did not respond to or experienced early relapse with a bortezomib-containing induction regimen and treated them with daratumumab, carfilzomib, lenalidomide and dexamethasone [144]. Longitudinal single-cell RNA-sequencing (scRNA-seq) was used to compare molecular differences between the 41 primary refractory and early relapsed patients, 11 healthy subjects, and 15 NDMM patients. PPIA was found to be upregulated in RRMM. Furthermore, CRISPR-Cas9 deletion of PPIA and ciclosporin inhibition of PPIA demonstrated good tumoricidal activity when used in combination with PI in PI-resistant cell lines [144]. PPIA inhibition may be a potential therapeutic approach for PI-refractory MM, and future studies are much anticipated.

## 14. Sec61 Translocon

Sec61 is a membrane protein complex that mediates secretory protein import into the ER. Sec61 inhibition prevents protein translocation into the ER and induces cytosolic degradation of newly synthesized proteins, which can eventually lead to cell death via continuous disruption of proteostasis [145]. Recent in vitro and in vivo studies explored the effects of a novel Sec61 inhibitor, mycolactone, on human MM cell lines and found that mycolactone causes defective immunoglobulin secretion and interferes with IL-6 mediated growth stimulation [146]. Mycolactone also exhibited a synergistic antimyeloma effect with bortezomib and lenalidomide, even in PI-resistant clones. Furthermore, mycolactone improved PI and IMiD bitherapy effectiveness in mouse models, including those engrafted with MM cell lines resistant to both PI and IMiD [145]. These results are encouraging and suggest that Sec 61 inhibitors could potentially be effective in the treatment of RRMM and should be investigated further.

## 15. Cyclin-Dependent Kinase 6 (CDK6)

CDK6 controls G1 to S cell cycle progression through regulation of the retinoblastoma (Rb) tumor suppressor protein and plays a role in the development of several cancers [147]. One study performed integrated global quantitative tandem-mass-tag (TMT)-based proteomic and phosphoproteomic analyses in MM samples and identified CDK6 upregulation to be a mechanism of IMiD resistance [148]. Furthermore, in vivo and in vitro analyses showed that CDK6 kinase inhibitor palbociclib restored IMiD sensitivity in resistance cell lines. Similarly, PROTAC-mediated CDK6 degradation reduced MM cell growth and demonstrated synergistic effects with IMiDs [148]. These results suggest that CDK6 may be a viable pharmacologic target to overcome IMiD resistance in MM.

## 16. Conclusions

Multiple myeloma remains an incurable disease despite numerous recent advances in treatment. Continued research of novel agents is needed to combat drug resistance to current therapies and produce long-lasting responses. Though recent advances in therapy have improved outcomes in myeloma significantly, treating multi-refractory patients with aggressive disease effectively remains a challenge. New targets and therapy combinations are hoped to improve outcomes for this population of patients. Moving forward, further research on the optimal sequencing of myeloma therapies is needed to determine the most appropriate way to administer the range of treatments currently available. Furthermore, continued advancement in our understanding and utilization of molecular information is needed to more accurately risk stratify and tailor myeloma therapy to each individual. With ongoing efforts to better understand and treat MM, we anticipate a future in which all myeloma patients can expect a prolonged survival and favorable quality of life.

## Figures and Tables

**Table 1:** Multispecific antibodies in multiple myeloma.

Target Antigen	Agent Name	NCT Number	Phase	N	Disease Status	Outcomes	CRS ICANS
BCMA × CD3	Teclistamab	NCT03145181 NCT04557098 (MajesTEC-1)	1/2	165	RRMM	ORR: 63%, ≥CR 39.4% mPFS: 11.3 mo	CRS: 72% ICANS: 3%
	Elranatamab	NCT04649359 (MagnetisMM-3)	2	123	RRMM	ORR: 61%, ≥CR: 35% mPFS: NR	CRS: 58% ICANS: 3%
	Linvoseltamab	NCT03761108 (LINKER-MM1)	1/2	117	RRMM	ORR: 71%, ≥CR: 29% mPFS: NR	CRS: 45%
	Alnuctamab	NCT03486067	1	73	RRMM	ORR: 54% MRD negative (10^−5^): 54% mPFS: 10.1 mo	CRS: 40% ICANS: 3%
	ABBV-383	NCT03933735	1	220	RRMM	ORR: 58%, ≥CR: 24% mPFS: 3.8/13.7/11.2 mo at 20/40/60 mg, respectively	CRS: 60% ICANS: 5%
BCMA × GPRC5D × CD3	JNJ-79635322	NCT05652335	1	ND	RRMM	ND	ND
BCMA × CD3 × Albumin	HPN217	NCT04184050	1	94	RRMM	ORR: 55% ≥VGPR: 40% among 12 mg and 24 mg weekly doses	CRS: 28% ICANS: 2%
GPRC5D × CD3	Talquetamab	NCT03399799 (MonumenTAL-1)	1/2	288	RRMM	ORR: 74%/73% ≥VGPR: 59%/57% mPFS: 7.5/11.9 in the QW and Q2W cohorts, respectively	CRS: 79%/75% ICANS: 11%/11%
		NCT05050097 (MonumenTAL-2)	1b	35	RRMM	ORR: 87%/83%, ≥CR: 60%/44% ≥VGPR: 87%/78% in the QW and Q2W cohorts, respectively	CRS: 74% ICANS: 6%
	Forimtamig (RG6234)	NCT04557150	1	51	RRMM	ORR: 71%, ≥CR: 24% VGPR: 29%	CRS: 78% ICANS: 6%
FcRH5 × CD3	Cevostamab	NCT05535244 (CAMMA 2)	1/2	ND	RRMM	ND	ND
		NCT05801939 (STEM)	2	ND	RRMM	ND	ND
CD38 × CD3	Igm-2644	NCT05908396	1	ND	RRMM	ND	ND
BCMA × CD38 × CD3	ISB-2001	NCT05862012	1	ND	RRMM	ND	ND

Abbreviations: ORR, overall response rate; CR, complete response; VGPR, very good partial response rate; mPFS, median progression-free survival; NR, not reached; ND, no data; RRMM, relapsed/refractory multiple myeloma; CRS, cytokine release syndrome; ICANS, immune effector cell-associated neurotoxicity syndrome.

**Table 2:** CAR T cell therapies in multiple myeloma.

Target Antigen	Agent Name	Cell Source	NCT Number	Phase	N	Disease Status	Outcomes	CRS ICANS
BCMA	Idecabtagene vicleucel	Autologous	NCT03361748 (KarMMa-1)	2	128	RRMM	ORR: 73%, ≥CR: 33% ≥VGPR: 52% mPFS/mOS: 8.8/19.4 mo	CRS: 84% ICANS: 18%
			NCT03651128 (KarMMa-3)	3	386	RRMM	ORR: 71%, CR: 39% mPFS: 13.3 mo	CRS: 88% ICANS: 15%
	Ciltacabtagene autoleucel	Autologous	NCT04133636 (CARTITUDE-2)	2	20	RRMM	ORR: 95%, CR: 75%, ≥VGPR: 85%	CRS: 85% ICANS: 20%
			NCT04181827 (CARTITUDE-4)	3	419	RRMM	ORR: 85%, ≥CR: 73%	CRS: 76% ICANS: 5%
	Ddbcma	Autologous	NCT04155749	1	13	RRMM	ORR: 100%, ≥CR: 75% VGPR: 8% mPFS: NR	CRS: 100% ICANS: 17%
	HBI0101	Autologous	NCT04720313	1	20	RRMM	ORR: 75%, ≥CR: 50%, VGPR: 25% mPFS: 160 days mOS: 308 days	CRS: 90% ICANS: 10%
	ARI0002h (cesnicabtagene autoleucel)	Autologous	NCT04309981 (CARTBCMA-HCB-01)	1/2	60	RRMM	ORR: 95%, ≥CR: 58% VGPR: 30% mPFS: 15.8 mo	CRS: 90% ICANS: 3%
	PHE885 (durcabtagene autoleucel)	Autologous	NCT04318327	1	46	RRMM	ORR: 98%	CRS: 96% ICANS: 22%
	Zevorcabtagene Autoleucel (CT053)	Autologous	NCT03975907 (LUMMICAR STUDY 1)	1	14	RRMM	ORR: 100%, ≥CR: 79% mPFS: 25.0 mo	CRS: 93% ICANS: 0%
			NCT03915184 (LUMMICAR-2)	2	14	RRMM	ORR: 100%, ≥CR: 40% VGPR: 10%	CRS: 86% ICANS: 7%
	Orvacabtagene Autoleucel (JCARH125)	Autologous	NCT03430011 (EVOLVE)	1/2	44	RRMM	ORR: 91%, ≥CR: 39% VGPR: 39%	CRS grade ≥ 3: 2%; ICANS grade ≥ 3: 4%
			NCT04960579	1	22	RRMM	ND	CRS: 14% ICANS: 4% GVHD: 0%
	BCMA-ALLO1	Allogeneic	NCT05066646 (FUMANBA-1)	1/2	103	RRMM	ORR: 96%, ≥CR: 78% MRD negative: 94%	CRS: 93% ICANS: 2%
	ALLO-715	Allogeneic	(NCT04093596) UNIVERSAL	1	43	RRMM	ORR: 71%, ≥CR: 25% VGPR 25%	CRS: 56% ICANS: 14%
	Equecabtagene autoleucel (eque-cel, CT103A)	Autologous	NCT04236011; NCT04182581	1	29	RRMM	ORR: 93%, sCR: 83% ≥VGPR: 90% MRD negativity (10^−4^–10^−6^): 100%	CRS: 86% ICANS: 0%
CD19/BCMA	GC012F	Autologous	NCT04555551	1	17	RRMM	ORR: 71%, ≥CR: 35% VGPR: 24%	CRS: 88% ICANS: 6%
GPRC5D	MCARH109	Autologous	NCT05016778 (POLARIS)	1	13	RRMM	ORR: 100%, ≥CR: 60% VGPR: 40%	CRS: 100% ICANS:0%
	OriCAR-017	Autologous	NCT04674813 (CC-95266-MM-001)	1	60	RRMM	ORR: 86%, CR: 38%	CRS: 84% ICANS: 11%
	BMS-986393 (CC-95266)	Autologous	ChiCTR2100048888	2	33	RRMM	ORR: 91%, ≥CR: 63% 12% VGPR	CRS: 76% ICANS: 9%
	anti-GPRC5D CAR T cells	Autologous	NCT03464916 (SOR-CART-MM-001)	1	9	RRMM	ORR: 33%, ≥CR: 0% SD: 33%	CRS: 22% ICANS: 11%
CD38	CAR2 Anti-CD38 A2 CAR T Cells	Autologous	NCT05007418 (DART-RRMM-101)	1	ND	RRMM	ND	ND
SLAMF7	SLAMF7 CAR T Cells	Autologous	NCT04499339 (CARAMBA-1)	1/2a	ND	RRMM	ND	ND
	CS1-BCMA CAR T cells	Autologous	NCT04662099	1	16	RRMM	ORR: 81%, sCR: 38% VGPR: 19% mPFS: NR	CRS: 38% ICANS: 0%

Abbreviations: ORR, overall response rate; CR, complete response; VGPR, very good partial response rate; SD: stable disease; mPFS, median progression-free survival; mOS, median overall survival; NR, not reached; ND, no data; RRMM, relapsed/refractory multiple myeloma; CRS, cytokine release syndrome; ICANS, immune effector cell-associated neurotoxicity syndrome.
